# Category learning in a recurrent neural network with reinforcement learning

**DOI:** 10.3389/fpsyt.2022.1008011

**Published:** 2022-10-25

**Authors:** Ying Zhang, Xiaochuan Pan, Yihong Wang

**Affiliations:** Institute for Cognitive Neurodynamics, East China University of Science and Technology, Shanghai, China

**Keywords:** category learning, stimulus-stimulus association, recurrent neural network, reinforcement learning, reward

## Abstract

It is known that humans and animals can learn and utilize category information quickly and efficiently to adapt to changing environments, and several brain areas are involved in learning and encoding category information. However, it is unclear that how the brain system learns and forms categorical representations from the view of neural circuits. In order to investigate this issue from the network level, we combine a recurrent neural network with reinforcement learning to construct a deep reinforcement learning model to demonstrate how the category is learned and represented in the network. The model consists of a policy network and a value network. The policy network is responsible for updating the policy to choose actions, while the value network is responsible for evaluating the action to predict rewards. The agent learns dynamically through the information interaction between the policy network and the value network. This model was trained to learn six stimulus-stimulus associative chains in a sequential paired-association task that was learned by the monkey. The simulated results demonstrated that our model was able to learn the stimulus-stimulus associative chains, and successfully reproduced the similar behavior of the monkey performing the same task. Two types of neurons were found in this model: one type primarily encoded identity information about individual stimuli; the other type mainly encoded category information of associated stimuli in one chain. The two types of activity-patterns were also observed in the primate prefrontal cortex after the monkey learned the same task. Furthermore, the ability of these two types of neurons to encode stimulus or category information was enhanced during this model was learning the task. Our results suggest that the neurons in the recurrent neural network have the ability to form categorical representations through deep reinforcement learning during learning stimulus-stimulus associations. It might provide a new approach for understanding neuronal mechanisms underlying how the prefrontal cortex learns and encodes category information.

## Introduction

Category is a fundamental concept in cognitive neuroscience. The literature has demonstrated that humans and animals can use categorical information quickly and efficiently to identify new objects, make inference and so on (1–3). For example, we could classify an animal as a dog on the basis of its physical characteristics, even the animal would be a new type of dog that we did not know before. And we could infer its basic properties that belong to the dog category commonly. There are two types of category in the literature: perceptual category and functional category. Objects sharing similar physical properties could be classified into a group as a perceptual category (4). A functional category indicates that its members that share no any physical similarity have the similar function, such as associating the same action or reward (5–7), etc. Many behavioral studies suggest that animals could form a functional category of a group of visual stimuli through training the matching-to-sample task (8, 9). In this task, some arbitrarily selected visual images (samples) are learned to associate with a common target image. After learning, it is found that animals could treat these visual images as equivalent stimuli, known as a functional category (10, 11). It is an important research topic in the literature of studying the category that how animals or the neuronal system could learn, represent and utilize category information.

Various experimental data, including fMRI studies, lesion studies, and neurophysiological studies, demonstrated that rather than a single brain area, many brain areas are involved in the categorical processing, such as the inferior temporal cortex, the prefrontal cortex (PFC), and the basal ganglia (12, 13). Different brain areas may have distinct contributions toward processing category-related information. Neurons in the inferior temporal cortex are more sensitive to perceptual features of stimuli than categorical relations (14–16). Neurons in the PFC can achieve the categorical distinction based on abstract rules (17). PFC neurons have stronger category coding ability than do inferior temporal cortex neurons in categorization tasks (18, 19), and neurons show more similar responses to stimuli belonging to the same category than to stimuli belonging to different categories (20, 21). In addition, the execution of actions in categorization decision-making tasks requires not only the involvement of the premotor cortex but also relevant functions of the basal ganglia to help the PFC complete the adjustment of strategies. Thus, it has been reported that the premotor cortex and the basal ganglia are also engaged in category learning (22–25). Although it is known that many brain areas perform different functional roles during category learning, the mechanism underlying how these areas cooperate to learn and encode the category is unclear. Therefore, we try to construct a network model to further understand the working mechanism of the neural system in a categorization decision-making task. In particular, the PFC plays essential roles in processing category information and we build the network model to mimic functional roles of the PFC in the categorization decision-making task.

Some theoretical models have been proposed to explain how the category is learned in the neural system (26–28). But most of models show categorical phenomena that are consistent with some behavioral results, without showing neural activity that encodes category information observed in the PFC or other brain areas (29, 30). Hinaut and Dominey constructed a neural network model of the PFC that demonstrated how categorization of behavioral sequences can be achieved through a recurrent system (31). Their model is a three-layer cortical neural network that is sensitive to the sequence. As a result, a few neurons in the three-layer model could identify each sequence and a few other neurons produce an explicit representation of the category to which sequences belong. However, this neural network model is able to discriminate categories by using supervised learning, which is not biologically plausible for animals learning in the decision-making task. Experimental studies have demonstrated that animals learn to perform specific tasks based on the reward feedback for taking action (32), known as reinforcement learning (RL).

A large number of studies have shown that a combination of artificial neural networks with RL could make the network model learn and storage items more efficiently and faster (33, 34). In particular, the RL has been used to understand neural mechanisms of association learning in the cerebral cortex (35, 36). In the RL framework, the agent takes action by trial and error, and then it can obtain rewards from the external environment. Its purpose is to maximize the expected amount of reward (37). Surprisingly, the recurrent neural network trained with repeated RL can mimic the complex behavior of animals observed in various decision-making tasks (38, 39). However, in most of these studies, the recurrent network was trained to learn stimulus-action associations or stimulus-reward associations in the tasks with single decision-making. Few studies have reported that the recurrent neural network with RL could be applied in category learning. We are interested in whether this type of model could learn the functional category for a group of stimuli through stimulus-stimulus associations in the tasks with multiple decision-makings.

In this study, we constructed a deep RL model that combines a recurrent neural network with RL to investigate how the category is learned in the network. On the one hand, this network model uses the gated recurrent unit network structure where neurons can regulate information transmission through gating mechanisms. On the other hand, this network model utilizes the actor-critic algorithm structure where neurons can update weights and biases through the policy gradient RL algorithm (40). Then, we investigate whether this model can mimic the behavior of monkeys and their neural activities in the PFC reported in a sequential paired-association task (41).

In the sequential paired-association task, this model needs to learn six stimulus-stimulus associative sequences in a similar way to train the monkey to learn this task. It was found that the model was able to successfully learn the six associative sequences at the end of the training, reproducing the choice behavior of the monkey observed in the task. Notably, we found two types of neurons in this model: one type primarily encodes information about individual stimuli; the other type mainly encodes category information of associated stimuli in one chain. The ability of these two types of neurons to encode information was enhanced during the learning process of this model. Our results suggest that the neurons in the recurrent neural network have the ability to form categorical representations through deep RL during learning stimulus-stimulus associations.

## Methods

### Neural network model

**Figure 1:**
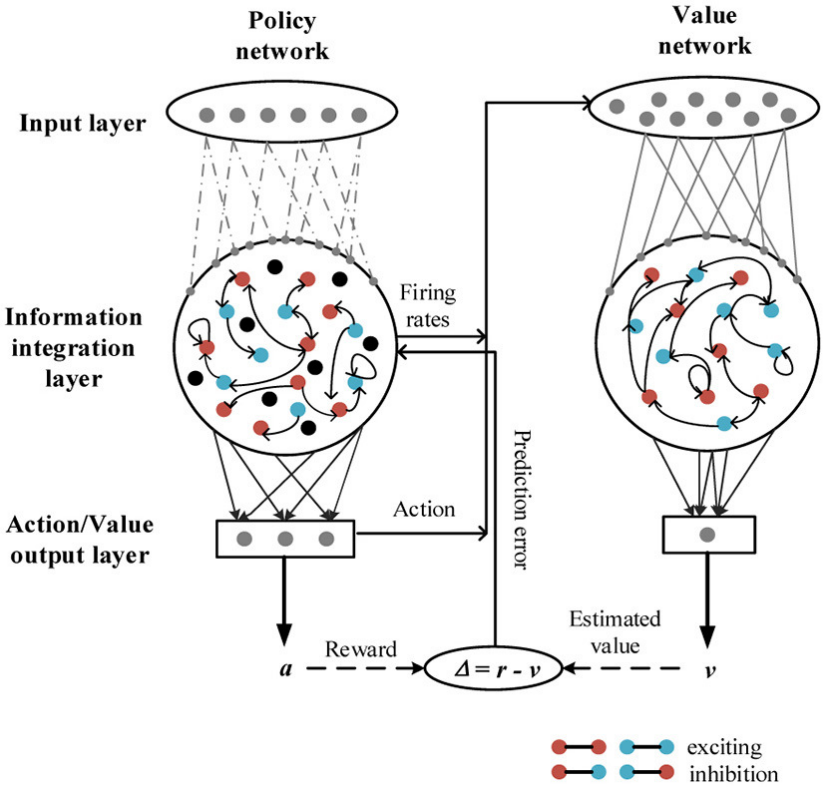
Structures of the neural network model. The deep RL neural network model, consisting of a policy network and a value network. In the policy network, sparse connections are made from the input layer to the information integration layer (IIL), among neurons in the IIL. Full connections are made from the IIL to the action output layer. In the value network, full connections are made among neurons in the input layer, the IIL, and the value output layer. In addition, in the IIL, red-red or blue-blue indicates excitatory connections between neurons; red-blue or blue-red indicates inhibitory connections between neurons; and black indicates no connection between neurons.

The deep RL network has been used to simulate stimulus-response associations or stimulus-reward associations in previous studies (38, 42). In this study, a new neural network based on the framework of the deep RL is proposed. The deep RL neural network model is composed of two parts: the policy network and the value network (Figure 1).

The policy network has three layers: the input layer, the information integration layer (IIL), and the action output layer. The number of neurons in the input layer is *N*_*p*_*in*_ = 11, and these neurons receive stimulus information from the external environment; the number of neurons in the IIL is *N*_*p*_ = 150, and these neurons can receive stimulus information from the input layer; the number of neurons in the action output layer is *N*_*p*_*out*_ = 3, and these neurons represent three actions: fixation, left and right choices in this study. The probability of connection from each neuron in the input layer to neurons in the IIL is *p*_0_ = 0.2; the probability of connection among neurons in the IIL is *p*_1_ = 0.1; the probability of connection from each neuron in the IIL to neurons in the action output layer is *p*_2_ = 1 (fully connected, see Table 1).

**Table 1:** Training parameters of the deep RL model.

**Parameter**	**Value**	**Description**
α	0.01	Learning rate
Δ*t*	20 *ms*	Time step
τ	100 *ms*	Time constant
*N*_*p*_*in*_	11	Number of neurons in the input layer (policy network)
*N*_*v*_*in*_	153	Number of neurons in the input layer (value network)
*N*_*p*_	150	Number of neurons in the IIL (policy network)
*N*_*v*_	100	Number of neurons in the IIL (value network)
*N*_*p*_*out*_	3	Number of neurons in the action output layer (policy network)
*N*_*v*_*out*_	1	Number of neurons in the value output layer (value network)
*p*_0_	0.2	Connection probability (policy network)
*p*_1_	0.1	Connection probability (policy network)
*p*_2_	1	Connection probability (policy network)
$\delta_{rec}^{2}$	0.01	Network noise
*N*_*trials*_	24	Number of trials for gradient update
*T*	121	Maximum time of per trial

The value network also has three layers. The number of neurons in the input layer is *N*_*v*_*in*_ = 153, and these neurons receive the firing rates of 150 neurons in the IIL and the action of 3 neurons in the action output layer of the policy network; the number of neurons in the IIL is *N*_*v*_ = 100, and these neurons can learn information from the policy network; the number of neurons in the value output layer is *N*_*v*_*out*_ = 1, and the neuron generates a predictive reward for each action. Here, full connections are made among neurons in the input layer, the IIL and the value output layer.

In this model, the policy network generates an action based on current stimulus and task conditions, and this model takes the action and receives an actual reward; the value network integrates neuronal firing rates in the policy network to output a predictive reward for the action. There is a reward prediction error between the actual reward and the predictive reward for the action, and the policy network adjusts the policy in time according to the error signal to minimize it.

In both the policy network and value network, the IILs have a recurrent connection structure with gated recurrent units (a gated recurrent unit is considered as a neuron). The gated recurrent unit includes an update gate and a reset gate, where the update gate is used to control the retained historical state information and receives new information about the candidate state, and the reset gate is used to control the dependence on historical state information for candidate information (43). In this way, information forms a dependency between different states of the transmission process. In this paper, the equations of the continuous-time gated recurrent unit network for the policy network are described in Equations (1)–(4), and the value network has similar equations for its gated units.


(1) $$\begin{array}{l} \phi_{i}\left(t\right)=\sigma (\sum_{j=1}^{N_{p}}W_{rec}^{\phi ,ji}x_{j}\left(t-1\right)\\ \;+\sum_{k=1}^{N_{p\_in}}W_{in}^{\phi ,ki}u_{k}\left(t\right)+b_{i}^{\phi }\left(t\right)),\;\left(i=1,\ldots ,N_{p}\right), \end{array}$$

(2) $$\begin{array}{l} \psi_{i}\left(t\right)=\sigma (\sum_{j=1}^{N_{p}}W_{rec}^{\psi ,ji}x_{j}\left(t-1\right)\\ \;+\sum_{k=1}^{N_{p\_in}}W_{in}^{\psi ,ki}u_{k}\left(t\right)+b_{i}^{\psi }\left(t\right)), \end{array}$$

(3) $$\begin{array}{l} h_{i}\left(t\right)=\left(1-\eta \phi_{i}\left(t\right)\right)h_{i}\left(t-1\right)\\ \;+\;\eta \phi_{i}\left(t\right)[\sum_{j=1}^{N_{p}}W_{rec}^{ji}\left(\psi_{j}\left(t\right)x_{j}\left(t-1\right)\right)+\sum_{k=1}^{N_{p\_in}}W_{in}^{ki}u_{k}\left(t\right)\\ \;+\;b_{i}\left(t\right)+\sqrt{2\eta^{-1}\delta_{rec}^{2}}\varepsilon ], \end{array}$$

(4) $$\begin{array}{lll} x_{i}\left(t\right) & = & {\left[h_{i}\left(t\right)\right]}^{+}. \end{array}$$

Here, we use the modified linear activation function [*x*]^+^ = max(0, *x*) as the output function of each neuron. Because the gated unit in GRU network is considered as the firing rate neuron, the value of its output function is defined as the firing rate of the neuron. The firing rate of each neuron in the IIL is non-negative. In addition, σ(*x*) = [1+exp(−*x*)]^−1^ as the output function of each gate [the update gate ϕ_*i*_(*t*) or the reset gate ψ_*i*_(*t*), (*i* = 1, …, *N*_*p*_), (*t* = 1, …, *T*)], ε is the Gaussian white noise with a mean of 0 and variance of 1, and $\delta_{rec}^{2}$ is used to control the size of this network noise. And *u*_*k*_(*t*) (*k* = 1, …, *N*_*p*_*in*_) is the input information of the *kth* neuron from the external environment at time *t*, *x*_*i*_(*t*) is the firing rate of the *ith* neuron at time *t*. $\eta =\Delta \frac{t}{\tau }$, Δ*t* is the time step, and τ is the time constant (Table 1), which is used to control the information dependency of gate recurrent units. $W_{rec}^{\phi ,ji}$ and $W_{rec}^{\psi ,ji}$ are the connection weights from the *jth* neuron to the *ith* neuron in the update gate and reset gate (44), respectively; $W_{in}^{\phi ,ki}$ and $W_{in}^{\psi ,ki}$ are the connection weights from the *kth* input neuron to the *ith* neuron in the update gate and reset gate, respectively; $b_{i}^{\phi }\left(t\right)$ and $b_{i}^{\psi }\left(t\right)$ are the bias of the update gate and reset gate, respectively. In addition, $W_{rec}^{ji}$ is the connection weight from the *jth* neuron to the *ith* neuron in the IIL; $W_{in}^{ki}$ is the connection weight from the *kth* neuron in the input layer to the *ith* neuron in the IIL; *b*_*i*_(*t*) is the bias of the *ith* neuron in the IIL.

Specifically, $x_{i}^{\pi }\left(t\right)$ is the firing rate of the *ith* neuron in the IIL of the policy network under the policy of π. Generally speaking, RL consists of five main elements: an agent, an environment, actions, states, and rewards. The agent first observes the external environment and receives the input information *u*_*t*_ (the *N*_*p*_*in*_ dimensional vector), and then according to the policy π_θ_(*a*_*t*_|*u*_*t*_) chooses an action *a*_*t*_ (the *N*_*p*_*out*_ dimensional vector). Here, the action output layer neurons generate an action based on the policy function:


(5) $$\begin{array}{l} z_{l}\left(t\right)=\overset{N_{p}}{\underset{i=1}{\sum }}W_{out}^{\pi ,il}x_{i}^{\pi }\left(t\right)\\ \;+b_{out}^{\pi ,l}\left(t\right),\left(l=1,\ldots ,N_{{p\text{\_}}_{out}}\right), \end{array}$$

(6) $$\begin{array}{lll} \pi_{\theta }\left(a_{t}=l|u_{t}\right) & = & \frac{e^{z_{l}\left(t\right)}}{\overset{N_{p_{out}}}{\underset{l=1}{\sum }}e^{z_{l}\left(t\right)}}. \end{array}$$

Where $W_{out}^{\pi ,il}$ (*l* = 1, …, *N*_*p*__*out*__) is the connection weight from the *ith* neuron in the IIL to the *lth* neuron in the action output layer of the policy network, $b_{out}^{\pi ,l}\left(t\right)$ is the bias of the *lth* neuron in the action output layer, *z*_*l*_(*t*) is the linear output function of the *lth* neuron in the action output layer, and the policy π_θ_(*a*_*t*_|*u*_*t*_) is the softmax function. The agent chooses an action based on the policy function through the random sampling method. That is to say, when the agent has very limited information about the external environment from observation, it cannot completely rely on the information to make a correct choice. However, the agent obtains a reward provided by the environment in the occasional event of taking action. In this case, an evaluation of the action by the value network can better guide the policy network to implement the adjustment of the policy. Here, the firing rate of the *mth* neuron in the IIL of the value network is $x_{m}^{v}\left(t\right)$ (*m* = 1, …, *N*_*v*_), and the neuron in the value output layer generates a predictive reward for the action based on the value function:


(7) $$\begin{array}{l} v_{\varphi }\left(x_{t}^{\pi },\;a_{t}\right)=\overset{N_{v}}{\underset{m=1}{\sum }}W_{out}^{v,m}x_{m}^{v}\left(t\right)+b_{out}^{v}\left(t\right). \end{array}$$

Where the firing rate $x_{t}^{\pi }$ (the *N*_*p*_ dimensional vector) of neurons in the IIL of the policy network and the action *a*_*t*_ (the *N*_*p*_*out*_ dimensional vector) of neurons in the action output layer as the input information of the value network. $W_{out}^{v,m}$ is the connection weight from the *mth* neuron in the IIL to the neuron in the value output layer, $b_{out}^{v}\left(t\right)$ is the bias of the neuron in the value output layer, and *v*_φ_ is the linear output information of the value output layer.

### Policy gradient reinforcement learning

In this model, the connection weights (*W*_*in*_, $W_{in}^{\phi }$, $W_{in}^{\psi }$, *W*_*rec*_, $W_{rec}^{\phi }$, $W_{rec}^{\psi }$, $W_{out}^{\pi }$, and $W_{out}^{v}$) and biases (*b*, *b*^ϕ^, *b*^ψ^, $b_{out}^{\pi }$, and $b_{out}^{v}$) of neurons are updated by the policy gradient RL algorithm during training (38). In this study, considering that the environmental state for the agent is not completely observable, we use a partially observable Markov decision process model, which is more suitable for the agent to learn in the state of limited information about the external environment. The partially observable Markov decision process model is discrete and finite (45). The continuous period is discretized through time steps, and the agent needs to observe the external environment and chooses an action at every time step. Setting the time ranges from 0 to time t, *I*_0:*t*_ is the historical information in the interaction process between the agent and the environment, including the states, observations, and actions, as follows:


(8) $$\begin{array}{l} I_{0:t}=\left(s_{0:t+1},u_{1:t},a_{0:t}\right). \end{array}$$

After the agent chooses an action *a*_*t*_ at the time t, it obtains a reward *r*_*t*+1_ at the next time *t*+1. In detail, when *t* = 0, the environment is in the current state *s*_0_ with the probability κ(*s*_0_), and the agent chooses an action *a*_0_ according to the policy π_θ_, where θ denotes the parameter, including the weights and biases of the policy network. When *t* = 1, the environment enters the new state *s*_1_ with the probability κ(_*s*_1_|*s*_0_, *a*0_), and the agent obtains a reward *r*_1_. Next, the agent observes the external environment and receives the input *u*_1_, and chooses an action *a*_1_ based on the new policy π_θ_(*a*_1_|*u*_1_) and obtains a reward *r*_2_. Thus, a process of the interaction between the agent and the environment is to keep repeating these steps until the end of each trial. In general, from the beginning to the end of each trial, the agent can rely on the policy π_θ_ at time t to choose an action *a*_*t*_ that eventually obtain the maximum expected value of the reward *R*(θ):


(9) $$\begin{array}{l} \;R\left(\theta \right)=E_{I}\left(\overset{T}{\underset{t=0}{\sum }}r_{t+1}\right). \end{array}$$

Where the T is the end time of each trial (Table 1), and the *E*_*I*_ is the expected calculation on the basis of the history *I*_0:*T*_ = (*s*_0:*T*+1_, *u*_1:*T*_, *a*_0:*T*_ ).

Our model utilizes the policy gradient method with an actor-critic algorithm structure when updating parameters. This approach uses the policy function and the value function for learning. Briefly, the actor takes action by adjusting the policy, which is the policy function; the critic evaluates each policy by predicting the reward of this action, known as the value function.

In order to update parameters of the policy network (actor) by the gradient descent method, an objective function is defined as follows:


(10) $$\begin{array}{l} \Gamma^{\pi }\left(\theta \right)=\frac{1}{N_{trials}}\overset{N_{trials}}{\underset{n=1}{\sum }}-R_{n}\left(\theta \right). \end{array}$$

Where the parameter θ includes the weights and biases of the policy network. Notably, when training the network model, we did not update parameters of the policy network in every trial; instead updating those after the completion of *N*_*trials*_ trials. This method makes learning process of the policy network more stable. In addition, we use the policy gradient algorithm to solve ∇_θ_*R*_*n*_(θ):


(11) $$\begin{array}{lll} \nabla_{\theta }R_{n}\left(\theta \right) & = & \overset{T}{\underset{t=0}{\sum }}\nabla_{\theta }\log\pi_{\theta }\left(a_{t}|u_{t}\right)\Upsilon \left(x_{t}^{\pi },a_{t}\right), \end{array}$$

(12) $$\begin{array}{lll} \Upsilon \left(x_{t}^{\pi },a_{t}\right) & = & \overset{T}{\underset{t=0}{\sum }}r_{t+1}-v_{\varphi }\left(x_{t}^{\pi },a_{t}\right). \end{array}$$

Here, the $\Upsilon \left(x_{t}^{\pi },a_{t}\right)$ is a reward prediction error value of the Temporal-Difference algorithm, which denotes the difference between the estimated value of the value function and the actual reward. This value can be used as an error signal to guide the policy network to learn. At the time *t*, $v_{\varphi }\left(x_{t}^{\pi }{,a}_{t}\right)$ is the linear output function of the value network, and $x_{t}^{\pi }$ is the firing rates of neurons in the IIL of the policy network.

In order to update parameters of the value network (critic) by the gradient descent method, an objective function is defined as follows:


(13) $$\begin{array}{lll} \Gamma^{v}\left(\varphi \right) & = & \frac{1}{N_{trials}}\overset{N_{trials}}{\underset{n=1}{\sum }}M_{n}\left(\varphi \right), \end{array}$$

(14) $$\begin{array}{lll} M_{n}\left(\varphi \right) & = & \frac{1}{T+1}\overset{T}{\underset{t=0}{\sum }}{\left[r_{t+1}-v_{\varphi }\left(x_{t}^{\pi },a_{t}\right)\right]}^{2}. \end{array}$$

Where *M*_*n*_(φ) is the mean square error, and the parameter φ includes the weights and biases of the value network. In the value network, the firing rates $x_{t}^{\pi }$ of neurons in the IIL of the policy network and the action *a*_*t*_ of neurons in the action output layer as its input information at time *t*, and its output information is a prediction value *v*_φ_ of the action. Here, we solve ∇_φ_*M*_*n*_(φ) by Backpropagation through the time algorithm (46). Finally, our model can learn dynamically based on the interaction of information between the policy network and the value network.

### Sequential paired-association task

**Figure 2:**
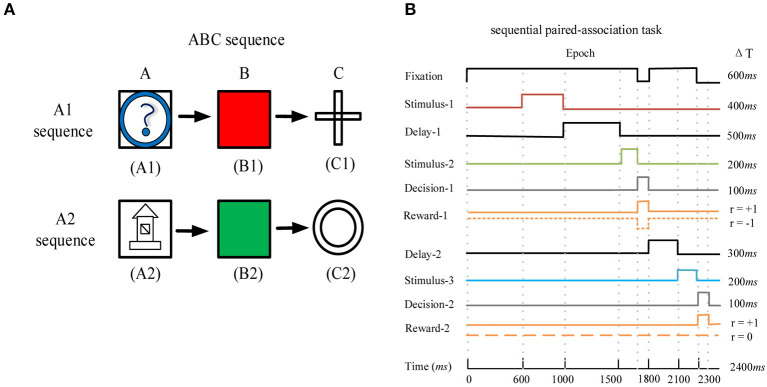
The sequential paired-association task and its task events. **(A)** The example of the ABC sequence learned by the monkey. The two correct stimulus-stimulus associative sequences are A1 → B1 → C1 and A2 → B2 → C2. **(B)** Timing of task events in a trial of the sequential paired-association task. The network model needs to fixate on the fixation spot during the stimulus and delay periods. It obtains a positive reward *r*_*t*+1_ = +1 for each correct choice during the two decision periods (Decision-1 and Decision-2). If this model makes a wrong choice in the first decision period, it will obtain a negative reward *r*_*t*+1_ = −1 and the current trial is terminated. If this model makes a wrong choice in the second decision period, it will not obtain a reward (*r*_*t*+1_ = 0) and the trial will end.

We used the deep RL model to learn the sequential paired-association task that has been performed successfully by the monkey (41). In this task, the monkey needed to learn two stimulus-stimulus associative sequences (Figure 2A). Here, the visual stimuli were six distinguishable pictures, which were divided into two associative sequences (A1 → B1 → C1 and A2 → B2 → C2). Figure 2B shows task events that are suitable for this model to learn. The maximum time of each trial is 2,400 ms (Figure 2B). At the beginning of each trial, the agent is required to fixate on the fixation spot for 600 ms. After that, the first stimulus A1 or A2 is presented for 400 ms. Following the first stimulus, there is a delay period of 500 ms. The agent continues fixating on the spot during the delay period. After the delay, the second stimuli B1 and B2 are presented simultaneously on the left and right positions. The left and right positions of the two stimuli are random. At this time, the agent is required to fixate on the spot for 200 ms. After the second stimuli is offset, the agent is given 100 ms to make the first choice (selection of B1 or B2 based on A1 or A2). If the first choice is wrong and the current trial is terminated. If the first choice is correct, the agent obtains a reward and the trial is to be continued. After the first correct choice, the agent is required to fixate on the spot for 300 ms. Then the third stimuli C1 and C2 are presented simultaneously, and the left and right positions of the two stimuli are random. At this time, the agent is required to fixate on the spot for 200 ms. After the third stimuli is offset, the agent is given 100 ms to make the second choice (selection of C1 or C2 based on B1 or B2). When the second choice is correct, the agent obtains a reward again and the trial is to end. The design of two associative sequences (A1-sequence and A2-sequence) allows the network model to select the target stimuli from the presentation of the target and distractor stimuli.

In the policy network, 11 neurons in the input layer denote the fixation, stimulus A1, stimulus A2, left stimulus B1, right stimulus B1, left stimulus B2, right stimulus B2, left stimulus C1, right stimulus C1, left stimulus C2, and right stimulus C2, respectively. In the sequential paired-association task, the fixation is labeled as a value of 1, the stimulus A1 or A2 is labeled as a value of 2, the stimulus B1 or B2 is labeled as a value of 3, and the stimulus C1 or C2 is labeled as a value of 4. The agent needs to take three actions (*N*_*out*_ = 3), and the three neurons in the action output layer are fixation (*a*_*t*_ = *F*), left (*a*_*t*_ = *L*), and right(*a*_*t*_ = *R*), respectively. We choose appropriate values for the number of neurons in the two IILs (*N* = 150 in the policy network and *N* = 100 in the value network) and their connection probabilities (see Table 1) in order to enable the model to learn the task successfully. We did not systemically analyze how changes of these super-parameters affect the model to learn the task. However, the combination of appropriate values of these super-parameters is important for the model to learn the task.

In general, the agent can choose left or right action only during two decision periods; and it must keep fixation during the stimulus period and the delay period. When the agent chooses a correct action in the first decision period, it obtains a positive reward *r*_*t*+1_ = +1; when the agent chooses a wrong action in the first decision period, it obtains a negative reward *r*_*t*+1_ = −1 and the trial is terminated. The agent obtains a positive reward *r*_*t*+1_ = +1 for the correct action or a reward *r*_*t*+1_ = 0 for the wrong action in the second decision period. If the agent does not make a choice (left or right) during the second decision period, it obtains a negative reward *r*_*t*+1_ = −1. During the stimulus period or the delay period, the agent chooses the fixation action to receive a reward *r*_*t*+1_ = 0; if the agent chooses a left or right action, it obtains a negative reward *r*_*t*+1_ = −1 and the trial is terminated.

The model is required to learn not only the ABC sequence (A1 → B1 → C1 and A2 → B2 → C2), but also the BCA sequence (B1 → C1 → A1 and B2 → C2 → A2) and the CAB sequence (C1 → A1 → B1 and C2 → A2 → B2). The three sequences have similar task events in a trial. We divided the six stimuli A1, A2, B1, B2, C1, and C2 into two groups, the A1-group (A1, B1, and C1) and the A2-group (A2, B2, and C2). The stimuli in the A1-group are associated each other in one chain and the stimuli in the A2-group are associated each other in another chain. When this model is trained, the three sequences (ABC, BCA, and CAB) appear randomly in the learning process, and the agent learns six stimulus-stimulus associative sequences in parallel.

In this task, we set the time constant τ to 100 ms, the time step Δ*t* to 20 ms, and the number of trials *N*_*trials*_ to 24, which denotes this network model updating parameters after 24 trials are completed (labeled as one iteration). In addition, when the network model completes 50 policy iterations, we test the network model with 800 trials to determine whether the policy is optimal. During the training process, the network model goes through the learning stage and testing stage alternately. The agent updates parameters through policy iterations in the learning stage, and the agent evaluates each policy without updating parameters in the testing stage. When the correct rate of choice (the ratio of correct trials to all trials) reaches 98% in the testing stage, we consider that the agent has found the optimal policy, which indicates that the network model can complete the task successfully.

The sequential paired-association task does not require the monkey to encode category information for the associated stimuli. Behaviorally, just memorizing each stimulus-stimulus association is sufficient for the monkey to perform the task successfully. However, it was reported that some prefrontal neurons encoded category information for the associated stimuli after the monkey learned the task (41, 47). We are interested in whether and how the network model forms categorical representations for associated stimuli during its learning of the sequential paired-association task.

### Category index and stimulus index

After this model learned stimulus-stimulus associations, we further examined the activity of 150 neurons in the IIL of the policy network. To characterize the response of each neuron, we calculate the category index for each of them during the first stimulus period (0–400 ms from the first stimulus onset). First, for each neuron, we calculate the absolute value of the firing rate difference of every two stimuli from the A1-group, which is denoted as *FD*_*A*1_. Similarly, we calculate the absolute value of the firing rate difference of every two stimuli from the A2-group, which is denoted by *FD*_*A*2_. Then, we calculate the mean firing rate difference of stimuli within a category for each neuron, which is denoted by *WCD*. The equations are as follows:


(15) $$\begin{array}{lll} WCD & = & \frac{FD_{A1}+FD_{A2}}{6}, \end{array}$$

(16) $$\begin{array}{lll} FD_{A1} & = & |x_{A1}-x_{B1}|+|x_{A1}-x_{C1}|+|x_{B1}-x_{C1}|, \end{array}$$

(17) $$\begin{array}{lll} FD_{A2} & = & |x_{A2}-x_{B2}|+|x_{A2}-x_{C2}|+|x_{B2}-x_{C2}|. \end{array}$$

Where || indicates the absolute value. *x*_*A*1_, *x*_*B*1_, and *x*_*C*1_ denote the firing rate of each neuron to stimuli in the A1-group during the first stimulus period; *x*_*A*2_, *x*_*B*2_, and *x*_*C*2_ denote the firing rate of each neuron to stimuli in the A2-group during the first stimulus period. After that, we also calculate the absolute value of the firing rate difference of each neuron between every two stimuli across the two groups. Thus, the difference value between two categories is denoted by *BCD*. The equations are as follows:


(18) $$\begin{array}{lll} BCD & = & \frac{FD_{1}+FD_{2}+FD_{3}}{9}, \end{array}$$

(19) $$\begin{array}{lll} {FD}_{1} & = & |x_{A1}-x_{A2}|+|x_{A1}-x_{B2}|+|x_{A1}-x_{C2}|, \end{array}$$

(20) $$\begin{array}{lll} {FD}_{2} & = & |x_{B1}-x_{A2}|+|x_{B1}-x_{B2}|+|x_{B1}-x_{C2}|, \end{array}$$

(21) $$\begin{array}{lll} FD_{3} & = & |x_{C1}-x_{A2}|+|x_{C1}-x_{B2}|+|x_{C1}-x_{C2}|. \end{array}$$

Finally, we define the category index according to *WCD* and *BCD*, which is denoted by *CI*, and it is given by:


(22) $$\begin{array}{l} CI=\frac{BCD-WCD}{BCD+WCD}. \end{array}$$

The range of *CI* is from −1 to 1. When the category index is negative, the neuron shows larger response-differences to stimuli within a category than to stimuli across the two categories. When the category index is positive, the neuron shows larger response-differences to stimuli across the two categories than to stimuli within a category.

Bootstrap test is used to determine whether the category index of each neuron is statistically significant from zero or not. We shuffled its firing rates among the six stimuli (A1, B1, C1, A2, B2, and C2) in the first stimulus period and calculated the category index based on the shuffled data. This process was repeated 500 times, generating a distribution of shuffled category indexes. The original category index value was deemed significant if it fell within the top or bottom 2.5% of the shuffled distribution (*p* < 0.05).

In addition, noting that some neurons show differential activity to stimuli from a category, we define the stimulus index for each neuron based on its firing rates to the three stimuli in the same category during the first stimulus period (48), denoted by *SI*, which is calculated as follows:


(23) $$\begin{array}{lll} SI & = & \frac{SI_{A1}+SI_{A2}}{2}, \end{array}$$

(24) $$\begin{array}{lll} SI_{A1} & = & \frac{{x\left(A1\right)}_{\max}-{x\left(A1\right)}_{\min}}{{x\left(A1\right)}_{\max}+{x\left(A1\right)}_{\min}}, \end{array}$$

(25) $$\begin{array}{lll} SI_{A2} & = & \frac{{x\left(A2\right)}_{\max}-{x\left(A2\right)}_{\min}}{{x\left(A2\right)}_{\max}+{x\left(A2\right)}_{\min}}. \end{array}$$

Where *x*(*A*1)_max_ denotes the maximum firing rate of each neuron to the three stimuli (A1, B1, and C1) in the A1-group during the first stimulus period, and *x*(*A*1)_min_ denotes the minimum firing rate to the three stimuli. *x*(*A*2)_max_ denotes the maximum firing rate of each neuron to the three stimuli in the A2-group during the first stimulus period, and *x*(*A*2)_min_ denotes the minimum firing rate to the three stimuli. The *SI* reflects response-differences to stimuli within a category, ignoring response-differences to stimuli across the categories. The range of *SI* is from 0 to 1, *SI* = 0 indicates that the neuron shows no differential activity to stimuli from a category, but it may have differential activity to stimuli from different categories.

## Results

Our model was performed using theano0.8.2 based on Python2.7 software in Windows 10 system, and the model was able to run successfully in learning the sequential paired-association task.

### Behavior performance of the network model

**Figure 3:**
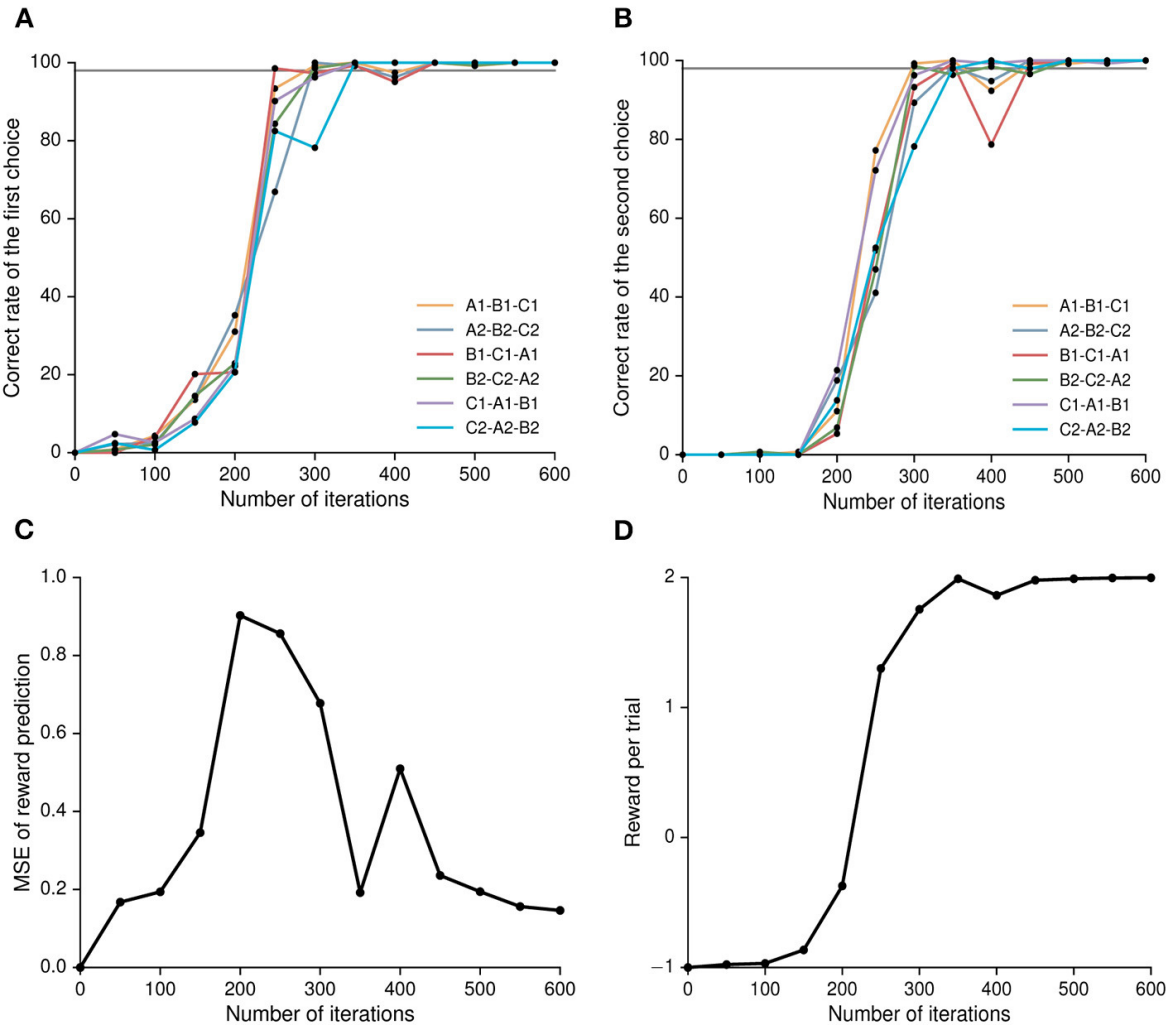
Behavior performance of the deep RL model. **(A)** Correct rate of the first decision period (the ratio of correct choice trials in the first decision period to all trial) for each stimulus-stimulus associations. **(B)** Correct rate of the second decision period (the ratio of correct choice trials in both decision periods to all trials) for each stimulus-stimulus associations. Here, the gray line denotes 98% of the target value. **(C)** The mean square error (MSE) of reward prediction for the network model in the second decision period (see Equation 14). Mean square error between the actual reward (based on the selected action in the policy network) and the predictive reward (estimated in the value network). **(D)** The reward obtained by the network model per trial.

The model was trained to learn the six stimulus-stimulus associations in parallel. In each trial, one of the six associations was inputted into the model. After 500 policy iterations, the network model could achieve the correct rate (the ratio of correct trials to all trials) of 98% in the two decision periods, indicating that it learned the sequential paired-association task (Figures 3A,B). It was worth noting that our network model needed to make two choices in each trial. In the early learning stage, the network model was trained to improve the correct rate of the first choice, the correct rate of the second choice was low. For example, the correct rate of the first choice and second choice were about 1.8 and 0% at the 50th policy iteration, respectively. When the network model increased gradually the correct rate of the first choice, it started to increase the correct rate of the second choice. At the 200th policy iteration, the correct rate of the first choice was about 25.4% and the correct rate of the second choice was about 12.6%. We found that from the 200th policy iteration, the mean square error (MSE) of reward prediction for the network model at the second choice decreased gradually during the training process (Figure 3C). It indicated that the predictive reward for the action estimated by the value network was getting closer to the actual reward. The result reflected that the network model could adjust the policy and choose a correct action in time through the feedback information provided by the error signal. The results suggested that our model could learn that the sequential paired-association task in different learning stages. Finally, this model was able to get the maximum reward in each trial (Figure 3D). The trained network model could reproduce the similar behavior of the monkey in the sequential paired-association task (41). It demonstrated that the model was able to learn stimulus-stimulus associative sequences.

### Various activity-patterns of neurons

**Figure 4:**
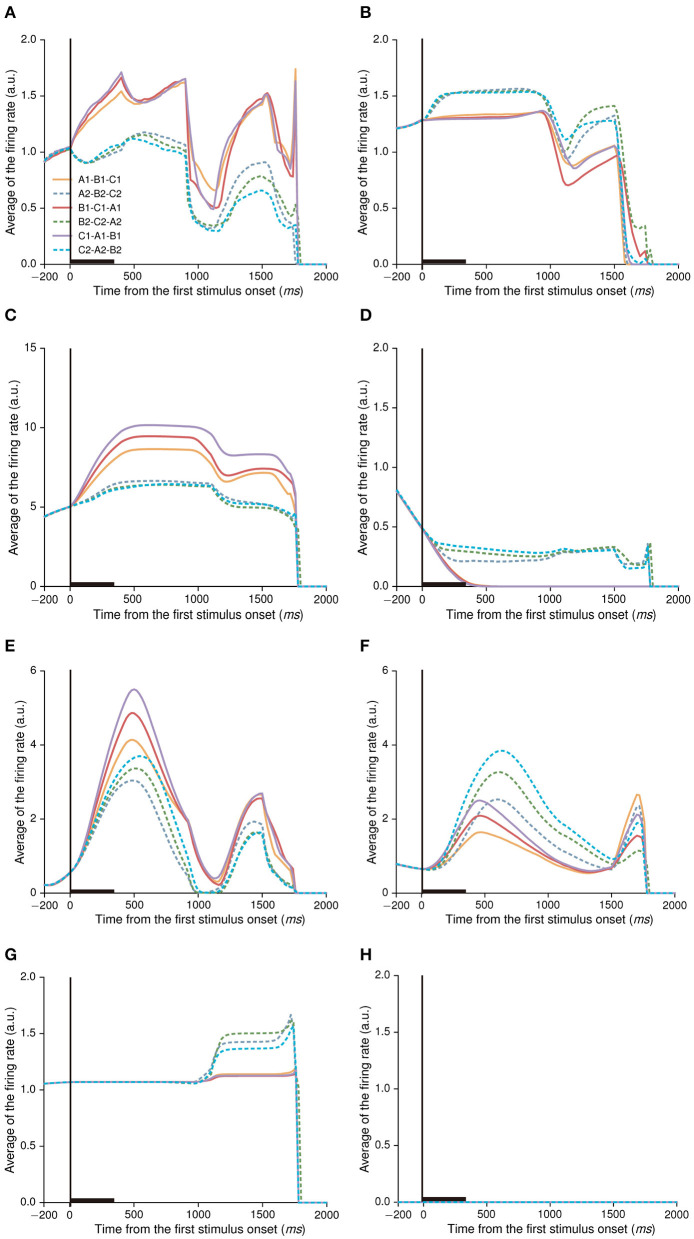
Various types of activity-patterns found in the IIL of the policy network after the model learned the task. Here, the black rectangle on the horizontal axis denotes the first stimulus period (0–400 ms from the first stimulus onset). During the first stimulus period, neurons show different activity in response to stimulus A1, A2, B1, B2, C1, and C2. Neural activity is sorted by the six stimulus-stimulus associations. If the firing rate in the first stimulus period increases compared to the baseline activity (a period of−200 ms-0 from the first stimulus onset), it indicates an excitatory response. If the firing rate decreases, it indicates an inhibitory response. The averaged firing rate of one neuron indicates that its firing rates are averaged across all trials. The same figure legends are used in **(A–G)**. **(A–D)** show example activity of category-neurons (*p* < 0.05, Bootstrap test), the category indexes of these neurons are 0.820, 0.872, 0.619, and 0.627, respectively. **(E–G)** show example activity of stimulus-neurons (*p* > 0.05, Bootstrap test), the category indexes of these neurons are 0.393, −0.135, and 0.416, respectively. **(H)** An example neuron showing no activity in the whole trial.

The output actions of this model demonstrated that it was able to correctly choose a target stimulus on the basis of the sample stimuli, indicating the model remembered stimulus-stimulus relations. How did neurons encode stimulus information and stimulus-stimulus relations to make a choice in our model? To investigate this issue, we further analyzed activity-patterns of neurons in the IIL of the policy network. Interestingly, neurons could produce various types of activity-patterns after our model learned the sequential paired-association task. During the first stimulus period (from 0 to 400 ms after the first stimulus onset), some neurons showed different responses to stimuli in the A1-group and the A2-group. For example, there are 19 neurons (19/150; 12.7%) produced excitatory activity to stimuli in the A1-group, and less activity to stimuli in the A2-group compared with the baseline activity (−200 to 0 ms from the first stimulus onset) (Figure 4A). Some neurons produced excitatory activity to stimuli in the A2-group and less activity to stimuli in the A1-group (Figure 4B), and the number of this type of neurons is 27 (27/150; 18%). About 14% (21/150) neurons produced excitatory activity to stimuli in the both A1-group and A2-group compared to the baseline activity (Figures 4C,E,F). In contrast, about 14.7% (22/150) neurons produced inhibitory activity to stimuli in the both A1-group and A2-group (Figure 4D). We also found that 16 neurons (16/150; 10.7%) showed no differential activity to stimuli in the both A1-group and A2-group (Figure 4G). Finally, about one third of neurons (45/150; 30%) kept silent during the whole trial (the firing rate of neurons was zero) (Figure 4H).

### Stimulus-neurons and category-neurons

Neurons in the IIL showed various types of activity-patterns. One important question is what kind of information these neurons encode in the model. We found that some neurons produced similar activity-patterns to the stimuli belonged to the same group, and differential activity-patterns to the stimuli belonged to different groups (see Figures 4A–D). The activity-patterns of these neurons were similar to those of PFC neurons observed in the sequential paired-association task (41). Many studies have demonstrated that PFC neurons can encode the category to which visual stimuli belong (49, 50). We hypothesized that neurons in this model could encode category information for each group of stimuli during stimulus-stimulus association learning.

**Figure 5:**
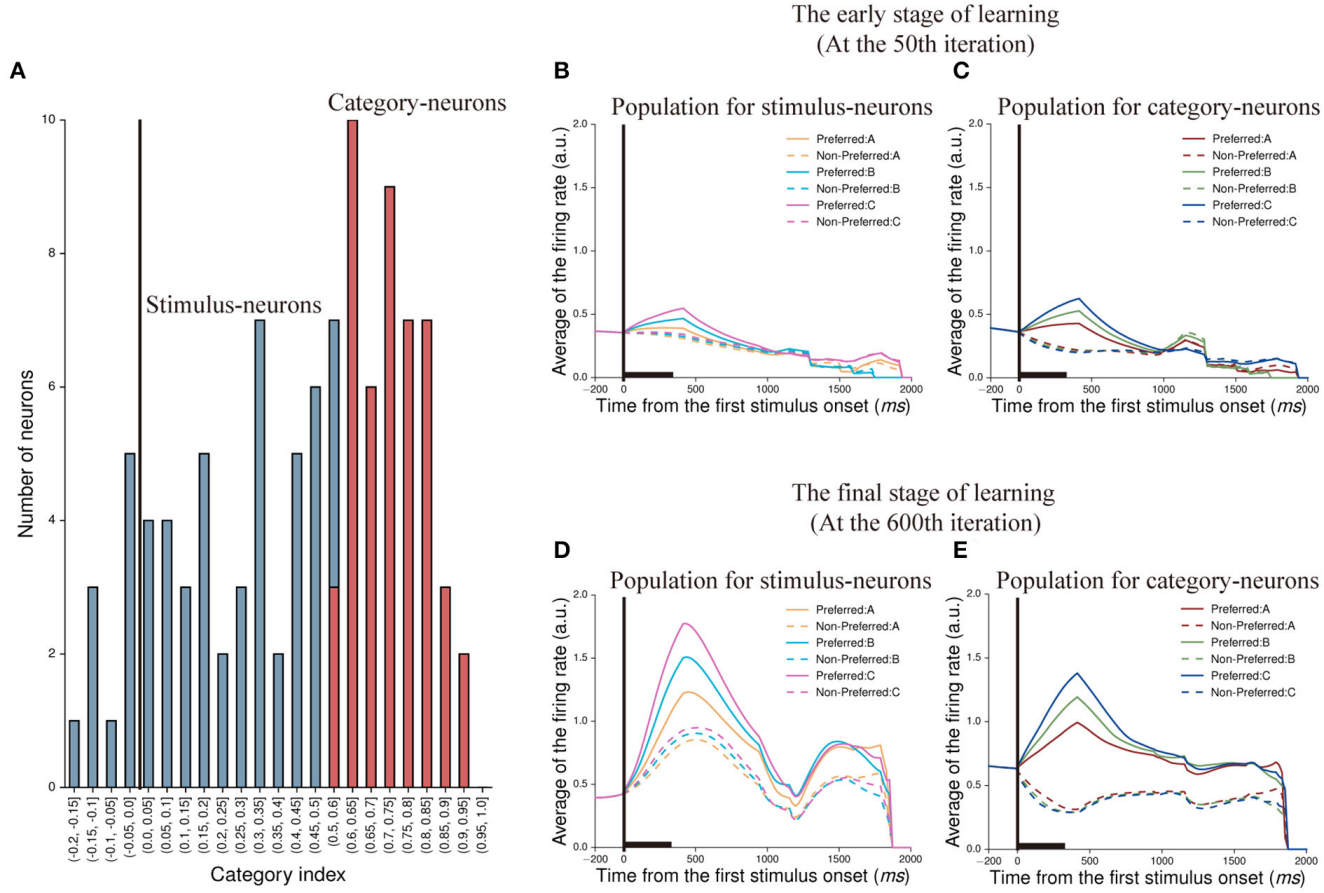
Classification of neurons and their population activity at two learning stages. **(A)** The distribution of category indexes. Here, blue bars indicate 58 neurons whose category indexes are not significant (*p* > 0.05, Bootstrap test), denoted as stimulus-neurons. And the range of category indexes for these neurons is from −0.2 to 0.6. Red bars indicate 47 neurons whose category indexes are significant (*p* < 0.05, Bootstrap test), denoted as category-neurons. And the range of category indexes for these neurons is from 0.5 to 1. **(B,C)** show population activity of stimulus-neurons **(B)** and category-neurons **(C)** in the early stage of learning (at the 50th iteration), respectively. The activity of each neuron is sorted by its preferred activity to the three paired stimuli (A1 vs. A2, B1 vs. B2, and C1 vs. C2) and then was averaged across neurons. **(D,E)** show population activity of stimulus-neurons **(D)** and category-neurons **(E)** in the final stage of learning (at the 600th iteration). The averaged firing rates shown in **(B,D)** are firing rates averaged across trials and across the stimulus-neurons. The averaged firing rates shown in **(C,E)** are firing rates averaged across trials and across category-neurons.

To demonstrate whether the neuron in our model was able to represent categorical information, we calculated the category index for each neuron in the first stimulus period. According to the definition of category index (see Section Methods), we calculated the category indexes of 105 neurons (excluding 45 no-response neurons shown in Figure 4H), and the range is from −0.2 to 1 (Figure 5A). We noted that some neurons had negative category indexes, indicating these neurons encode less category information, whereas some neurons had positive category indexes encoded more category information. In order to determine whether the category index of individual neuron is significantly different from zero, the bootstrap method was used (see Section Methods). The results showed that 58 neurons in this IIL had an insignificant category index (*p* > 0.05) and the mean category index of these neurons was 0.243. We thought that these neurons could not identify the category to which the stimulus belongs, but encoded stimulus identity. These neurons are referred to as stimulus-neurons. In addition, 47 neurons had a significant category index (*p* < 0.05) and the mean category index of these neurons was 0.731. These neurons primarily encoded category information, denoted as category-neurons. It suggested that there were individual neurons having the ability to encode category information in our model.

Next, we created population histograms for stimulus- and category-neurons at different learning stages, respectively. In the sequential paired-association task, the stimulus-neurons and the category-neurons produced different activity to stimuli. We found that when this model was in the early learning stage (at the 50th iteration) of the task, the neurons of both populations could show activity differences between the preferred and non-preferred stimuli during the first stimulus period and the first delay period. However, from the second stimulus period, these activity differences gradually disappeared for the both types of neurons (Figures 5B,C). When this model was in the final learning stage (at the 600th iteration) of the task, both stimulus-neurons and category-neurons show stronger activity to preferred stimuli than that to non-preferred stimuli in the whole trial (Figures 5D,E). The results indicated that although the information encoded by neurons would decay with time in the process of transmission, the neurons would gradually enhance the storage capacity of information and form working memory through learning.

**Figure 6:**
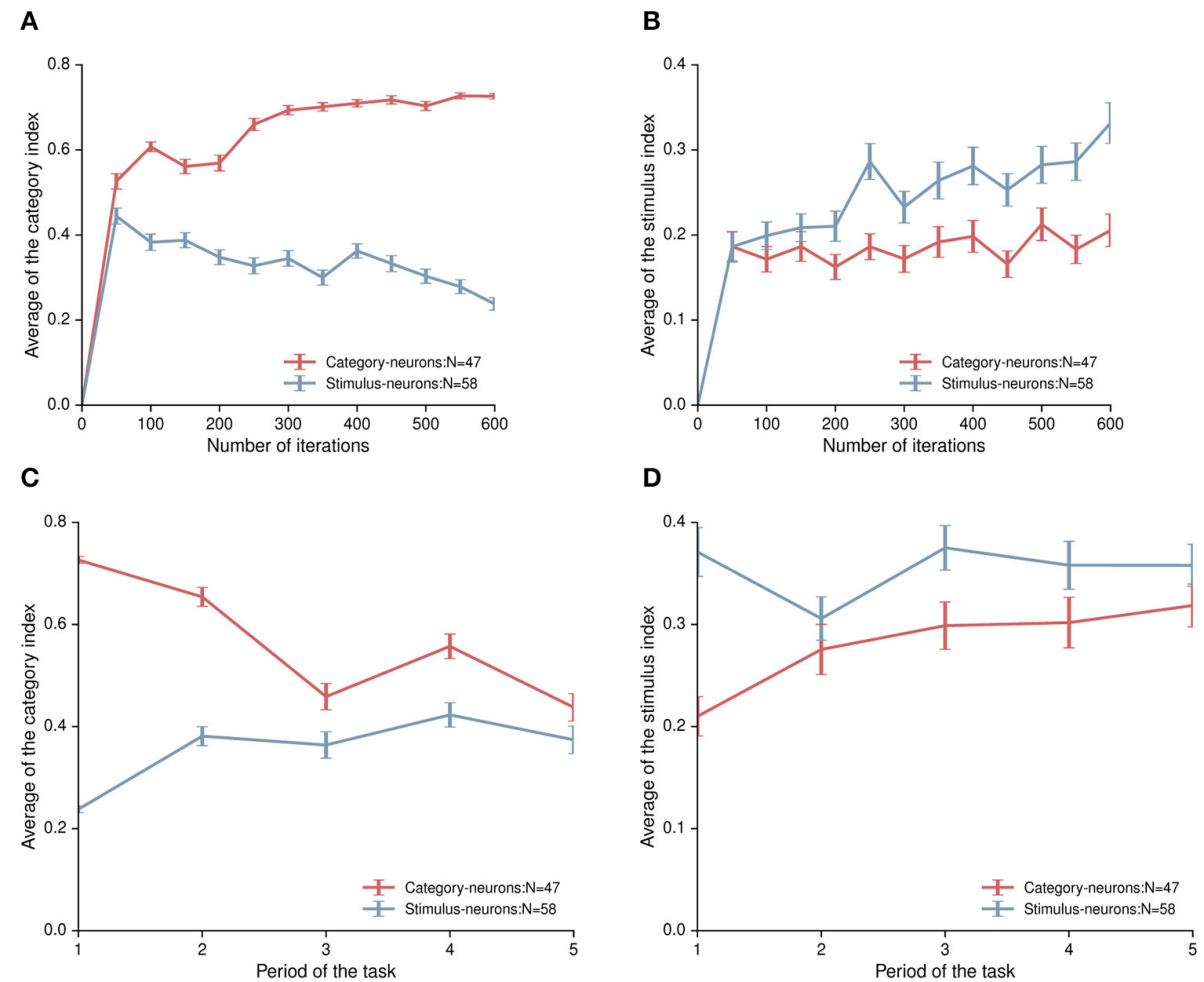
The category index and the stimulus index of category-neurons and stimulus-neurons. **(A)** The time course of the category index for category-neurons (the red curve) and stimulus-neurons (the blue curve) during the network model learning the task. **(B)** The time course of the stimulus index for category-neurons (the red curve) and stimulus-neurons (the blue curve) during the network learning the task. **(C)** The category indexes for category-neurons (the red curve) and stimulus-neurons (the blue curve) in five different task periods after the model learned the task. **(D)** The stimulus indexes for category-neurons (the red curve) and stimulus-neurons (the blue curve) in five different task periods. The number “1,” “2,” “3,” “4,” and “5” in the horizontal coordinates indicate the first stimulus period, the first delay period, the second stimulus period, the second delay period, and the third stimulus period, respectively.

In order to quantitatively measure activity-changes during the learning process, we calculated the category index for each stimulus- and category-neuron in each testing stage, respectively. The mean category index of the stimulus-neurons decreased gradually, and the mean category index of the category-neurons increased gradually during the learning process of the task (Figure 6A). It meant that category-neurons enhanced the ability to encode category information through learning; while stimulus-neurons did not exhibit the characteristic of enhanced ability to encode category information.

Second, we quantitatively characterize the ability of both types of neurons to encode stimulus information during the learning process. We computed the stimulus index for each neuron to denote response-differences to within-category stimuli (see Section Methods). The mean stimulus index of 58 stimulus-neurons increased gradually, and the mean stimulus index of 47 category-neurons kept relatively stable during the learning process of the task (Figure 6B). The result of the Mann-Whitney U test showed that there was a significant difference in the ability of two populations to discriminate within-category stimuli in the final learning stage (*p* = 0.018). For stimulus-neurons, although their ability for category coding decreased, their ability for stimulus coding obviously increased.

It was obvious that the ability of these two types of neurons to encode information was enhanced during the learning process of this model, and their activity also changed in different task periods. We further analyzed the characteristics of neurons encoding information in different task periods. Interestingly, the category-neurons show the strongest ability to encode category information in the first stimulus period, and the ability decreased after the first stimulus period. Even though, the mean category index of category-neurons was higher than that of the stimulus-neurons in each task period (Figure 6C). The stimulus-neurons showed the strongest ability to encode stimulus information in the first stimulus period, and this ability also decreased after the first stimulus period. But in each task period, the mean stimulus index of stimulus-neurons was higher than that of the category-neurons (Figure 6D).

**Figure 7:**
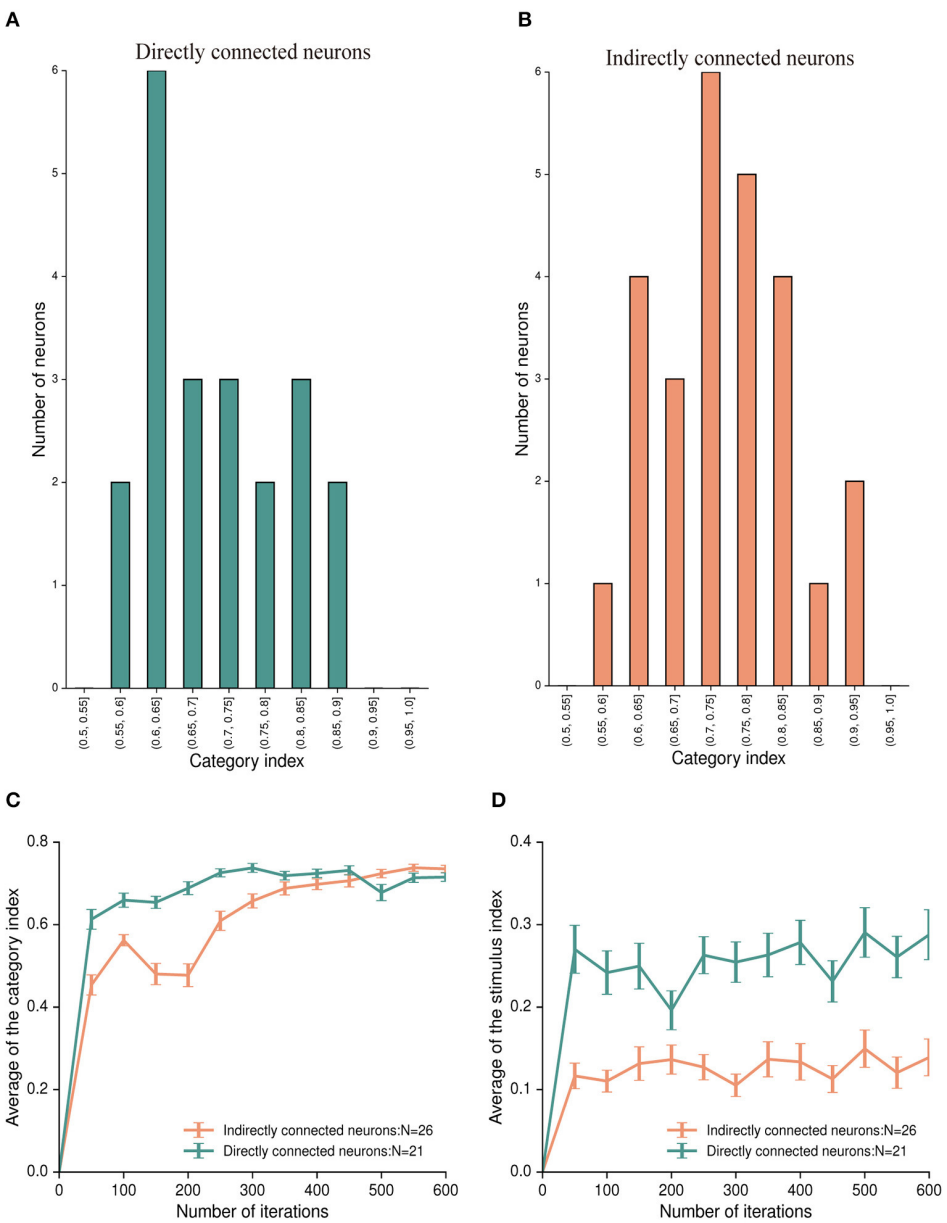
Category index and stimulus index for two types of category-neurons: directly connected neurons and indirectly connected neurons. **(A)** The distribution of category indexes of 21 category-neurons, which have direct connections from the input layer. The range of category index for these neurons is from 0.5 to 0.9. **(B)** The distribution of category indexes of 26 category-neurons, which have no direct connections from the input layer. The range of category index for these neurons is from 0.5 to 1. **(C)** The time course of category index for the directly connected neurons (the aquamarine curve) and the indirectly connected neurons (the salmon curve), respectively. **(D)** The time course of the stimulus index for the directly connected neurons (the aquamarine curve) and the indirectly connected neurons (the salmon curve), respectively.

Although the stimulus-neurons and category-neurons may play different roles in this model, we found that category-neurons encoded not only category information but also stimulus information (see Figure 5E, category-neurons could discriminate the three preferred stimuli). One question is whether the stimulus information found in the category-neurons is directly influenced by external stimuli? It was worth noting that in the policy network, sparse connections were used between neurons in the input and IILs. And only some neurons in the IIL directly received stimuli from the input layer (these neurons are denoted as directly connected neurons), while other neurons did not (those neurons that do not receive direct projections from the input layer as indirectly connected neurons). We analyzed the activity differences of the two groups of directly and indirectly connected neurons. In the first stimulus period, 54 (54/150; 36%) neurons in the IIL were directly connected with neurons in the input layer. Within them, 21 (21/54; 38.9%) neurons were identified as the category-neurons. And their mean category index was 0.715 (Figure 7A). In addition, 96 (96/150; 64%) neurons did not receive direct connections from the input layer. Among these 96 neurons, 26 (26/96; 27.1%) of them were identified as the category-neurons. And their mean category index was 0.745 (Figure 7B). The two groups of category-neurons had similar distributions of category indexes (see Figures 7A,B). Furthermore, we found that the two groups of neurons showed different learning curves of the category index (Figure 7C). The mean category index of directly connected neurons increased quickly in the early learning stage (at the 50th iteration) and changed slightly at later learning stages (from the 300th iteration to the 600th iteration). The mean category index of indirectly connected neurons increased obviously at different learning stages (from the 50th iteration to the 600th iteration). In the final learning stage (at the 600th iteration), the two groups of neurons showed similar category indexes (Mann-Whitney U test, *p* = 0.250).

We further calculated the stimulus indexes for the two groups of directly connected neurons, and indirectly connected neurons (Figure 7D). The mean stimulus index of directly connected neurons was significantly higher than that of indirectly connected neurons. The result of the Mann-Whitney U test showed that external stimuli directly affected the ability of category-neurons to discriminate between stimuli (*p* = 0.002). It indicated that the ability of neurons to encode category information during category learning was not directly influenced by external stimuli; whereas the ability of neurons to encode stimulus information was directly influenced by external stimuli.

### Weight analysis of neurons in the network

It was found that the neurons in this model were capable of stimulus coding and category coding. This model updated weights during learning the sequential paired-association task. In general, the synaptic plasticity of neurons is crucial in constructing models (51, 52). This is because the information is exchanged between neurons with the help of synaptic connections, and the type of synapses (excitatory or inhibitory synapses) and their values affect the activity of neurons (53). At the computational level, excitatory synapses increase firing rates of neurons, while inhibitory synapses diminish their firing rates. So how does the interaction of excitatory and inhibitory synapses affect the learning process of neurons? Therefore, we discussed the connection weights of neurons.

**Figure 8:**
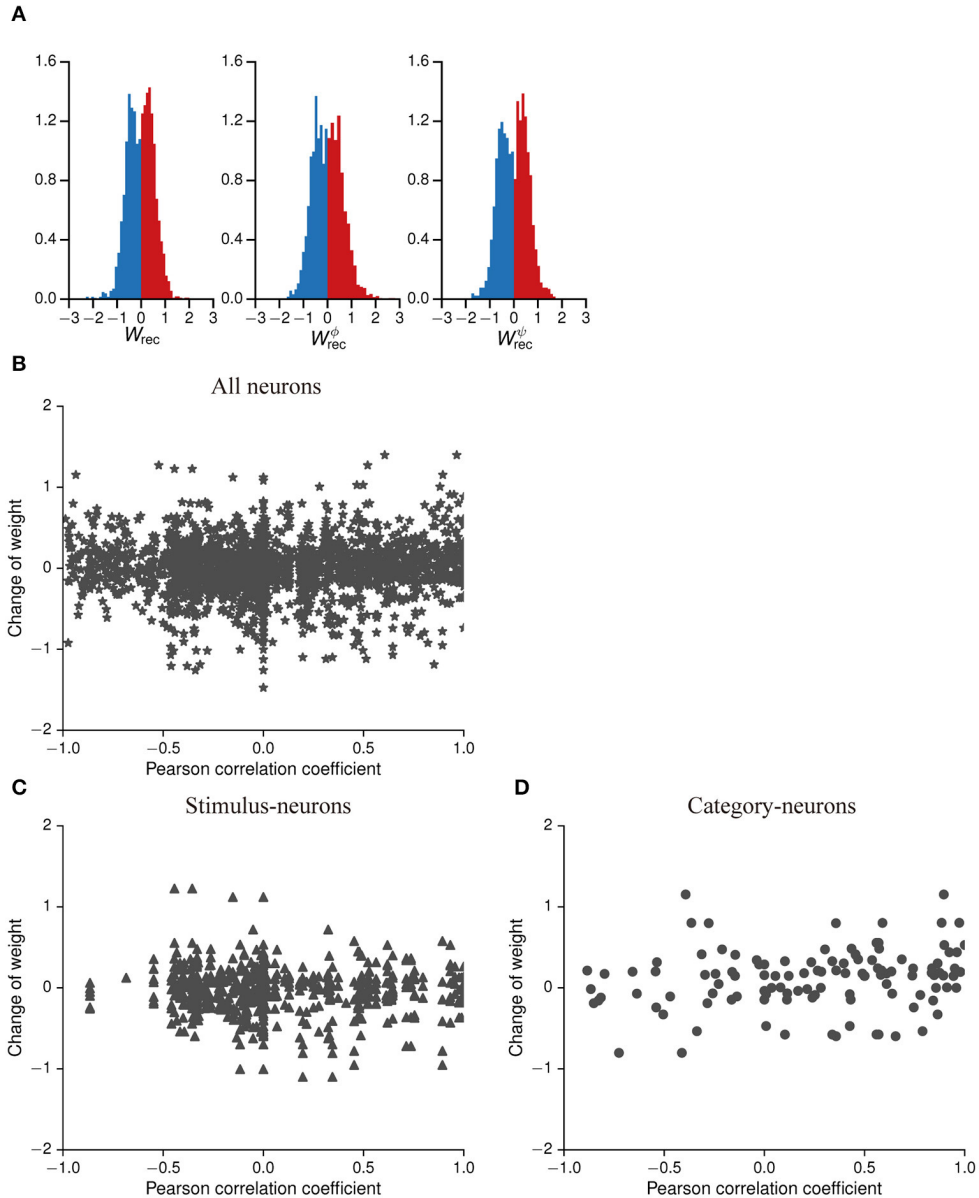
Distributions of weights in the policy network and the correlation analysis between activity-patterns and weight changes. **(A)** Frequency distribution histogram about the connection weights of neurons in the IIL of the policy network. The dark red bars denote excitatory weights, which are positive, and the dark blue bars denote inhibitory weights, which are negative. Left panel: weights of among neurons in the recurrent network; middle panel: weights of the update gates; right panel: weights of the reset gates. **(B–D)** show correlation analysis between the activity-pattern similarity of each pair of neurons (Pearson correlation coefficient) and their weight change. **(B)** All pairs of neurons that have connection in the IIL. **(C)** Pairs of connected neurons are selected only from stimulus-neurons. **(D)** Pairs of connected neurons are selected only from category-neurons.

In the policy network of this model, the neurons in the input layer were sparsely connected to the neurons in the IIL with the probability of 0.2. Most neurons in the IIL could not directly receive the stimuli from the external environment. Here, among neurons in the IIL were sparsely connected with the probability of 0.1, and the neurons indirectly learned the stimuli from the external environment through information transmission. When this model was trained, we recorded the connection weights of neurons in the IIL, where positive values were excitatory weights and negative values were inhibitory weights. The connection weights of these neurons were Gaussian distribution (Figure 8A), and a balance mechanism was formed between excitatory weights and inhibitory weights.

Next, we asked a question whether the weight change between two connected neurons was correlated to the similarity of their activity-patterns. We would expect that neurons having more similar activity-patterns had stronger connection weights to form connection structures in the IIL during the learning process. To understand this problem, we selected every pair of connected neurons, and calculated the Pearson correlation coefficient of their activity-patterns in the first stimulus period. In addition, we also calculated the weight change (the difference between the weight at the end of training and the initial weight). Figure 8B shows scatter plots of the Pearson correlation coefficient and the weight change for all pairs of neurons. There is no correlation between them. Specifically, we used the same method to calculate the Pearson correlation coefficient and the weight change for the stimulus-neurons (Figure 8C) or for the category-neurons only (Figure 8D). Even within the same type of neurons, the similarity of their activity-patterns is not correlated with their weight changes. Although category-neurons were able to identify the category to which the stimuli belong, their activity-patterns were not directly influenced by the weights. As we know, the structure of recurrent neural networks is extremely complex. The neurons are not only involved in updating weights during the learning process but also influenced by other factors, such as the decay of information and the importance of information, which meant that neurons produce similar activity performance as the result of the synergistic effect of multiple variables.

### Neural activity related to action selection

**Figure 9:**
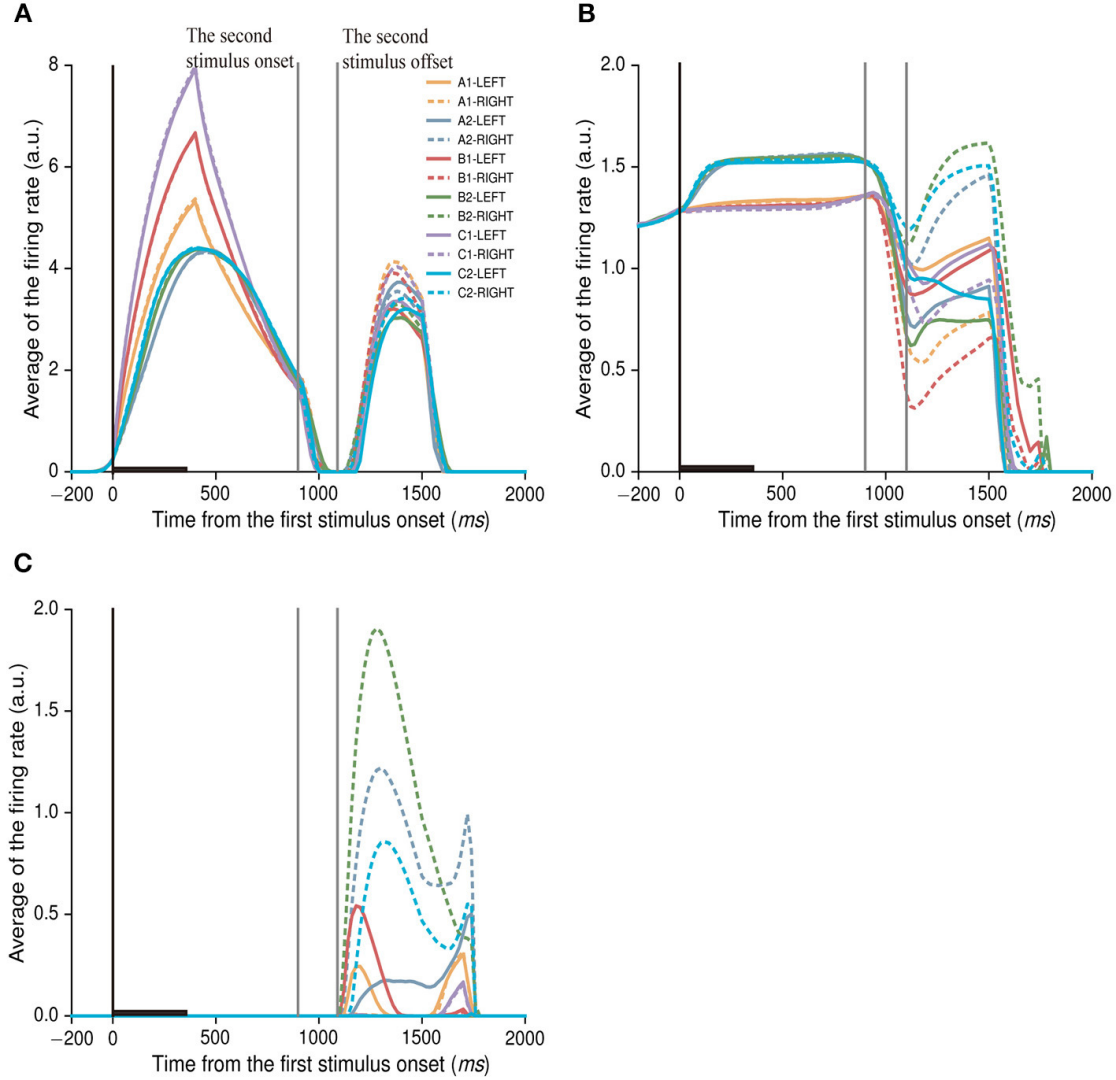
The activity of neurons related to action choices. Here, the two gray lines indicate the second stimulus period, after the second stimulus period, the network model chooses an action (left or right) during the first decision period. The activity of each neuron is aligned on the first stimulus onset, and is sorted with stimulus-position conditions. The same figure legends are used in **(A–C)**. **(A)** An example neuron shows only stimulus-related activity in the first stimulus and delay periods, no differential activity to actions (or positions). **(B)** An example of neuron shows not only category-related activity in the first stimulus and delay periods, but also activity related stimulus-action combinations after the second stimuli offset. **(C)** An example of neurons shows only stimulus-action related activity after the second stimuli offset, no stimulus-related activity either in the first stimulus period or in the delay period. The averaged firing rate of one neuron indicates its firing rates that are averaged across all trials.

Till now, we focused on analyzing neuronal activity in the first stimulus period, and found that majority of neurons encoded stimulus and category information. During the first stimulus period, the model had not to make a choice of action (left or right), there was no choice-related activity in this period. In the first decision period after the second stimuli offset, the model had to make a left or right choice. How was the choice-related information encoded in the IIL? In order to investigate this issue, we aligned neural activity at the first stimuli onset and sorted the activity into stimulus-position conditions (12 stimulus-position conditions, see Figure 9). We mainly found three types of activity-patterns in the first decision period (Figure 9). The first type of neurons showed no differential activity in response to the left position and right position in the first decision period, but showed differential activity to stimuli in the first stimulus and delay periods (Figure 9A). This type of neurons may encode only stimulus-related information, no action-related activity. There were 21 neurons (21/150; 14%) that were classified into this type of neurons in the IIL. The second type of neurons could simultaneously encode stimulus-related information in the first stimulus and delay periods and stimulus-action combined information in the first decision period (Figure 9B). This type of neurons encoded information from pure stimulus-related information to stimulus-action information at different task periods. The number of this type of neurons was 75 (75/150; 50%) in the IIL. These neurons may contribute to transfer stimulus information into action information. The third type of neurons showed stimulus-action combined information only, no difference in response to the stimulus (Figure 9C). This neuron mainly discriminated between left-right actions. There were only 7 neurons found in the IIL. This type of neurons mainly contributed to action selection in the model. In addition, one third of neurons (47/150; 30.9%) showed no response during the whole trial (see Figure 4H; the firing rate of neurons was zero). The IIL neurons were able to encode stimulus information and position information, which were passed to neurons in the action output layer. The connection weights between neurons in the IIL and action output layer were dynamically adjusted during the training of this model. Finally, our model could learn the task.

## Discussion

In this study, we demonstrated that the recurrent neural network using RL could learn the six stimulus-stimulus associative sequences. Through the trial-and-error method, the model first learned correct actions in the first choice and then in the second choice, a similar learning method was observed for the monkey in the same task. Various types of neural activity were found in the IIL in the first stimulus period. Some neurons encoded information of individual stimulus, and some neurons encoded category information of a group of stimuli that were associated together. These types of activity were also reported in the primate PFC in the sequential paired-association task (41). Actually, the stimulus-stimulus association task did not require the monkey and the model to form a categorical representation. However, some neurons in the PFC and in the IIL of the model did encode category information for associated stimuli. The categorical representation might help the monkey or the model accelerating the learning process. For example, the categorical representation of an associated stimuli enables them to easily select the target stimulus from a same category of the sample stimuli, without the requirement to memorize the specific target stimulus that is associated with the sample. Some neurons in the IIL also showed heterogenetic activity in different task periods (see Figure 9B). This type of heterogenetic activity-pattern was often observed in the PFC in different cognitive tasks (54). We found that almost half of model-neurons encoded stimulus (or category) information in the first stimulus and delay periods and encoded stimulus-position combined information in the decision periods. A few neurons encoded only stimulus-position combined information after the second stimuli offset. We did not find neurons that encoded pure position information (left or right action). The model learned stimulus information and then transferred it into stimulus-position combined information, and neurons in the action output layer integrated stimulus-position combined information to generate a correct action.

Many studies, including behavioral, neurophysiological, and fMRI experiments, suggest that the brain system learns categorical representations with a two-stage model of category learning (10, 20, 55). In the first stage, the sensory systems identify and represent stimulus information based on its physical properties (56). In the second stage, the associative brain areas encode meanings of a group of stimuli to form categorical representations. In our model, two types of neurons were found: stimulus-neurons and category-neurons. These neurons encode different aspects of task information, implying the model may learn category information with two different representations. Category-neurons encoded not only category information but also some stimulus information (see Figure 5E). Although those indirectly and directly connected neurons had the same level of category-indexes in the final learning stage, the former learned category information was slower than the latter (see Figure 7C). And the indirectly connected neurons also had significantly smaller stimulus-indexes than the directly connected neurons. These results indicate that inputs from the input layer may affect neurons in the IIL to learn category information. Further weight analysis suggested that stimulus-neurons or category-neurons did not form cluster or hierarchical structures in the IIL. The similarity of activity-patterns of a pair of neurons did not correlate to their weight changes (see Figure 8). In the current model, synaptic connections from the input layer to the IIL and within the IIL were sparse and random. A pre-determined connection structure in the IIL may help the model to learn representing stimulus information, category information and action information in a hierarchical manner.

The recurrent neural networks with the RL algorithm have been widely used to simulate behavior and neural activity of animals in cognitive tasks (57, 58). In this framework, our model is trained with the RL in a way similar to that the animals learn the cognitive task with trial and error. Model-neurons in the recurrent network appear complex and heterogenetic activity-patterns (see Figure 4). The RL algorithm plays a critical role in our model. Notably, the RL algorithm has a rich historical research background in machine learning (59–61). It has been reported that some brain areas, such as the PFC, the basal ganglia, and the dopamine system, implement RL to interact with the environment. Biologically, the PFC is critical in implementing strategies (62), and its neurons encode information about actions by adjusting strategies (63, 64). In addition, the PFC and the basal ganglia are interconnected to form a recurrent structure (65, 66). Specifically, the PFC and the striatum are closely linked (67). The dopamine is released from VTA (68) and SNc to the striatum and then acts on the PFC, and the information conveyed by dopamine is taken as the prediction error of the reward (69, 70), then the PFC adjusts the strategy based on the error signal from the striatum. Thus, the PFC is regarded as the policy network and the striatum is regarded as the value network (71).

It has been found that the brain areas, including the visual cortex, PFC, parietal cortex, premotor cortex, and basal ganglia are involved in the process of category learning (12, 21), in which the PFC neurons are more capable of encoding category information (72). Interestingly, in this model, we found that some model-neurons could encode category information for a group of stimuli that are associated each other in one chain, consistent with the finding that some PFC neurons encoded category information of those associated stimuli in the sequential paired-association task (10). While we don't know how exactly the PFC or the neuronal system to learn and form categorical representations for associated stimuli in the task, one possible way suggested by our current model is that the PFC and its related brain areas may implement deep RL to encode category information during learning the task.

It is well known that PFC neurons encode not only stimulus and category information but also reward information. For example, in the sequential paired-association task with an asymmetric reward schedule (47), PFC neurons showed strong responses to the stimulus that was associated with a large reward; when the reward amount was reversed for the same stimulus (large reward became small reward), these neurons responded slightly to the stimulus. This result demonstrated that the neural activity was affected by the amount of reward. We tried to make this model to learn the sequential paired-association task with an asymmetric reward schedule, but the activity-pattern of neurons was not influenced by the reward reversal. The possible reason is that we did not take into account the reward amount as a model parameter in this model. The reward amount is just considered as an error signal to modify connection weights in the policy network. Therefore, neurons in the IIL do not encode reward or stimulus-reward information. Remarkably, environmental stimuli as input information affect the neural activity in the model. If model-neurons receive different reward amounts as input information, their activity may reflect reward and stimulus-reward combined information, and this model might be able to complete the sequential paired-association task with an asymmetric reward schedule. This issue should be further investigated.

The simulated results in this study also demonstrate that the network model is able to encode categorical information efficiently. Hinaut and Dominey reported that some neurons in a three-layer recurrent neural network with randomly-initiated weights could encode the categorical structure of a set of behavioral sequences without the requirement to modify the weights (31). But the three-layer recurrent neural network did not encode category information efficiently, the percentage of such categorical neurons was very low (0.4% of total neurons) (31). Our model shows efficient ability to encode category information, almost one-third (47/150) of neurons have category-selectivity. However, there are still some limitations in the current model. For example, although it was found that neurons need the capacity of working memory to learn the sequential paired-association task, there was lack of detailed description of the working mechanism by which neurons store memory information. It is known that the brain has extremely complex neuronal circuits that are involved in category learning. Our model is a single-layer recurrent neural network, which has a relatively simple network structure. In the future, combination the long-short-term memory network (73, 74) with the asynchronous actor-critic algorithm (75, 76) should be included to construct models with multilayer recurrent structures to simulate functions of category-related neuronal circuits.

In summary, we use the framework of deep reinforcement learning (the recurrent network + reinforcement learning) to build the novel network model that is trained to learn the sequential paired-association task. This task requires the network model to make two sequential choices to learn stimulus-stimulus associations in one trial. Our new findings in this study are that the network model can perform the task correctly after being trained with the trial-and-error method, indicating that the model has the ability to learn the complex structure of the task, not just to learn simple stimulus-action or stimulus-reward associations as reported in previous studies (38, 42). More importantly, we found stimulus-neurons and category-neurons in the IIL of the policy network. These two types of neurons represent different aspects of task parameters, and their ability to encode category and stimulus information was strengthened during the learning process. The model neurons in the IIL show heterogenetic activity to encode information of the stimulus, category, action and their combinations. These responsive properties of neurons in the IIL are similar to activity-patterns observed in the primate PFC in the same task (41, 47), indicating the IIL could mimic functions of the PFC in the categorization tasks. The simulation results indicate that the recurrent neural network could learn the categorical representation for a group of stimuli in the matching-to-sample task (stimulus-stimulus associations) using the RL algorithm, without additional requirements such as the network structure, prior knowledge or specific categorical rules. Our results might provide a new way for understanding neuronal mechanisms underlying how the brain system learns category information.

## Data availability statement

The original contributions presented in the study are included in the article/supplementary material, further inquiries can be directed to the corresponding author.

## Author contributions

XP, YZ, and YW contributed to conception, design of the study, and wrote sections of the manuscript. YZ wrote the codes, performed simulating the model, and wrote the first draft of the manuscript. YZ and XP analyzed the data. All authors contributed to manuscript revision, read, and approved the submitted version.

## Funding

This study was supported by the National Natural Science Foundation of China (Nos: 11972195, 12172132, 11802095) and Natural Science Foundation of Shanghai (No: 19zr1473100).

## Conflict of interest

The authors declare that the research was conducted in the absence of any commercial or financial relationships that could be construed as a potential conflict of interest.

## Publisher's note

All claims expressed in this article are solely those of the authors and do not necessarily represent those of their affiliated organizations, or those of the publisher, the editors and the reviewers. Any product that may be evaluated in this article, or claim that may be made by its manufacturer, is not guaranteed or endorsed by the publisher.
